# Evaluating the competency of ChatGPT in MRCP Part 1 and a systematic literature review of its capabilities in postgraduate medical assessments

**DOI:** 10.1371/journal.pone.0307372

**Published:** 2024-07-31

**Authors:** Oliver Vij, Henry Calver, Nikki Myall, Mrinalini Dey, Koushan Kouranloo

**Affiliations:** 1 Guy’s Hospital, Guy’s and St Thomas’ NHS Foundation Trust, Great Maze Pond, London, United Kingdom; 2 Basel, Switzerland; 3 British Medical Association Library, BMA House, Tavistock Square, London, United Kingdom; 4 Centre for Rheumatic Diseases, Denmark Hill Campus King’s College London, London, United Kingdom; 5 Department of Rheumatology, University Hospital Lewisham, London, United Kingdom; 6 School of Medicine, Cedar House, University of Liverpool, Liverpool, United Kingdom; Federal University of Paraiba, BRAZIL

## Abstract

**Objectives:**

As a large language model (LLM) trained on a large data set, ChatGPT can perform a wide array of tasks without additional training. We evaluated the performance of ChatGPT on postgraduate UK medical examinations through a systematic literature review of ChatGPT’s performance in UK postgraduate medical assessments and its performance on Member of Royal College of Physicians (MRCP) Part 1 examination.

**Methods:**

Medline, Embase and Cochrane databases were searched. Articles discussing the performance of ChatGPT in UK postgraduate medical examinations were included in the systematic review. Information was extracted on exam performance including percentage scores and pass/fail rates.

MRCP UK Part 1 sample paper questions were inserted into ChatGPT-3.5 and -4 four times each and the scores marked against the correct answers provided.

**Results:**

12 studies were ultimately included in the systematic literature review.

ChatGPT-3.5 scored 66.4% and ChatGPT-4 scored 84.8% on MRCP Part 1 sample paper, which is 4.4% and 22.8% above the historical pass mark respectively. Both ChatGPT-3.5 and -4 performance was significantly above the historical pass mark for MRCP Part 1, indicating they would likely pass this examination.

ChatGPT-3.5 failed eight out of nine postgraduate exams it performed with an average percentage of 5.0% below the pass mark.

ChatGPT-4 passed nine out of eleven postgraduate exams it performed with an average percentage of 13.56% above the pass mark. ChatGPT-4 performance was significantly better than ChatGPT-3.5 in all examinations that both models were tested on.

**Conclusion:**

ChatGPT-4 performed at above passing level for the majority of UK postgraduate medical examinations it was tested on. ChatGPT is prone to hallucinations, fabrications and reduced explanation accuracy which could limit its potential as a learning tool. The potential for these errors is an inherent part of LLMs and may always be a limitation for medical applications of ChatGPT.

## Introduction

Artificial intelligence (AI) has transformed the way we approach a huge number of industries and tasks ranging from consumer products to computer programming, as well as medicine [1,2]. Previously, the development of clinical AI models required significant time and resources with highly domain-specific training data [3]. However, with the release of ChatGPT by OpenAI in November 2022, the application of AI to many industries, including medicine, became far more accessible [4]. Within the medical field, ChatGPT has potential applications in medical education, clinical reasoning and research [5–7].

ChatGPT is a large language model (LLM) powered by OpenAI’s Generative Pre-trained Transformer(GPT)-3.5 or -4 and was seen as a major breakthrough in AI [8]. Previous iterations of AI language models were largely based on sequential style neural networks, such as recurrent neural networks (RNN) and long short-term memory (LSTM) neural networks [9–12]. With the introduction of the transformer architecture by Vaswani et al. in 2017, this has been widely adopted to develop LLMs such as GPT [13]. The key innovations with transformer architecture are two-fold. Firstly, they can process sequences non-sequentially. This means they do not ‘forget’ tokens far back in a sequence and the next word prediction considers the whole context not just the last few words. Secondly, self-attention, which weighs each token differently depending on the context, without which, the model could be basing its prediction on irrelevant information [13]. This ultimately means that LLMs can generate novel sequences never previously observed by the model [1,14]. As LLMs are trained on a vast data set, this allows ChatGPT to perform a wide array of tasks without the need for any additional specific training. It is able to generate computer code and analyse data, translate between languages, write discharge summaries and answer examination questions [8,15–18]. This had led to ChatGPT being tested on several medical examinations since its release to the public [1,19–26]. Most notably, ChatGPT-3.5 was shown to operate at or near pass level in the infamous United States Medical Licensing Examination (USMLE) [1]. GPT-4 was then released on 14^th^ March 2023, which can reportedly solve more difficult problems with greater accuracy due to a broader general knowledge and problem-solving ability [27]. Indeed, with the upgraded GPT-4, ChatGPT’s USMLE step 1 score increased from 64.5% to 88% [1,28].

Following its success on the USMLE, ChatGPT-4 has now passed numerous national undergraduate medical licensing examinations including those from the Australia, China, Iran, Japan, Korea, Peru, Saudi Arabia and the UK [19–26]. ChatGPT’s performance has also been tested on several postgraduate medical examinations.

We wanted to further evaluate the ability of ChatGPT-3.5 and -4 to undertake postgraduate medical exams without any additional training. We conducted a systematic literature review (SLR) of ChatGPT’s performance in UK postgraduate medical assessments to analyse the level of knowledge it is able to accurately report. To consolidate this review, we tested ChatGPT-3.5 and -4 on Part 1 of the Membership of the Royal College of Physicians (MRCP Part 1) examination and included these results in the analysis. MRCP Part 1 is a postgraduate UK medical qualification accessible to doctors with a minimum of 12 months experience in medical employment, that tests candidates using multiple choice questions [29]. MRCP Part 1 was selected firstly, as it has been demonstrated to be a reliable examination [30]. Secondly, because it is an internationally recognised postgraduate medical assessment attempted by around 30% of UK medical graduates which forms a critical part of career progression for aspiring physicians in the UK [31].

## Methods

### Systematic Literature Review (SLR)

This SLR was undertaken in accordance with the Cochrane Handbook and reported as per the Preferred Reporting Items for Systematic Review and Meta-Analysis [32,33].

The review question was: What is the performance of ChatGPT on UK postgraduate medical examinations?

Databases were searched for the performance of ChatGPT on UK postgraduate medical examinations. Outcomes from the paper included: iteration of ChatGPT used, pass/fail rates, percentage score on the examination and factors influencing exam outcome.

### Search strategy, databases and study selection

To ensure comprehensive coverage, indexing terms (MeSH, applicable to Medline and Cochrane, and Emtree headings on Embase) in addition to keyword searching were used. The full search strategy is available in the supplementary material.

Medline, Embase and Cochrane databases were searched for articles discussing the performance of ChatGPT-3.5 and -4 on UK postgraduate medical examinations from their conception until 24^th^ January 2024. The search was restricted to English-language articles. Eligible articles included: observational studies, qualitative studies, and randomised control trials.

Full-length articles were uploaded onto Rayyan (www.rayyan.ai) with duplicates removed. Articles that met the inclusion criteria were examined by one author at abstract and full paper stage, with a 20% validity screening. Information was extracted on exam performance including percentage scores and pass/fail rates as well as factors that influenced exam outcome.

### Assessment of performance of ChatGPT-4 in MRCP Part 1

The MRCP UK Part 1 sample questions, accessible via The Royal College of Physcians’ website, were used [34]. This consisted of 197 questions each with 5 multiple choice options from A to E. These questions, along with their options A to E, were compiled into a single text file. This text file was inserted as individual questions into ChatGPT-3.5 and as a single text into ChatGPT-4 to mimic an examination paper. Answers given by ChatGPT were recorded and marked against the correct answers provided by the MRCP UK mark scheme. The performance of ChatGPT-3.5 and -4 were marked as a total score out of 197 and as a percentage. The examination paper was entered into ChatGPT-3.5 and -4 four times and the results recorded. Four repeats were used as a study evaluating ChatGPT response consistency found no statistically significant difference between ChatGPT-3.5 or -4 performance following three rounds of questions [35]. The Shapiro-Wilk and Levene’s tests were applied to the data with all P values >0.5, suggesting the data was normally distributed with equal variance. An independent samples T-test was used to test the significance between the historical pass mark of MRCP Part 1 and the average scores of ChatGPT-3.5 and -4. MRCP Part 1 questions were categorised into factual recall or complex reasoning question types and the number of each question type that ChatGPT got incorrect was counted. A two-proportion Z test was used to calculate if the number of each question category ChatGPT got incorrect was significant when compared with the number of these questions within the MRCP Part 1 sample questions.

To assess whether MRCP (UK) Part 1 sample questions were in ChatGPT training data or if the models were working out answers for themselves, we applied an algorithm known as memorisation effects Levenshtein detector (MELD) [36]. To our knowledge this is the only algorithm used to detect if LLMs were trained on data that is inputted, and has been applied to other studies with similar methodology [37–39]. The proposed data set is split into halves with the first half inserted into the LLM and the LLM output matched to the second half of the data set. If the LLM output and second half of the data set share a 95% overlap or more, it is likely that the LLM was trained on that data set. MELD was implemented using the algorithm in the appendix of the paper that introduced this approach with all 197 sample questions and answers tokenised and split into pairs [36]. The full code for the application of MELD is available on GitHub (https://github.com/calvh3/gpt_tester). GPT-4’s API was set with a temperature of 0. Following the implementation of MELD, none of GPT-4’s completions had greater than a 95% match with the MRCP (UK) Part 1 sample paper questions, indicating that it is unlikely ChatGPT-4 was trained on this data set.

## Results

### Systematic literature review

**Fig 1:**
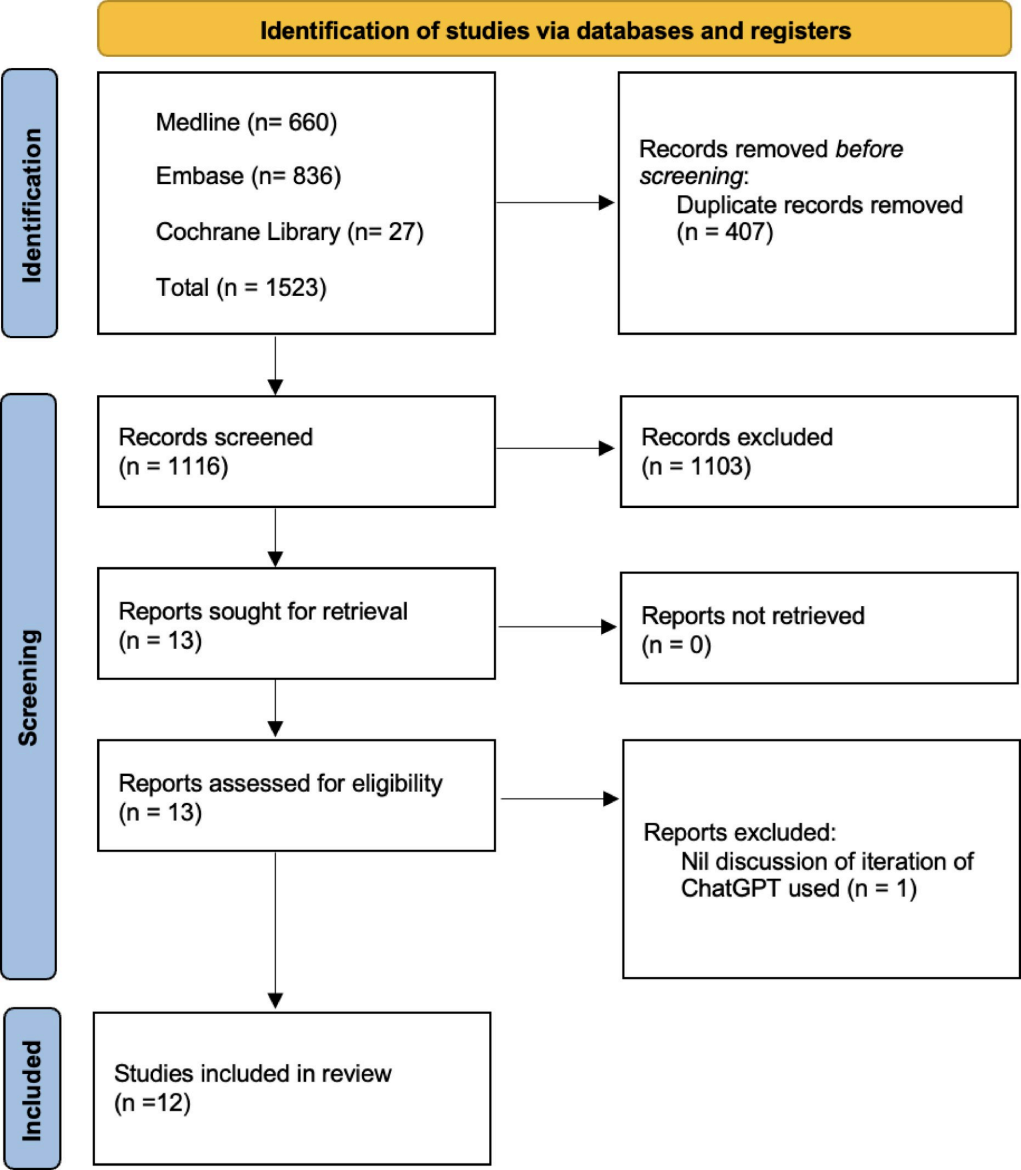
Flow diagram of stages of systematic literature review. Cochrane Library encompasses library of: Systematic reviews; systematic review protocols; controlled clinical trials.

Initially 1116 articles were retrieved with 12 included (Fig 1). This gave a total of 12 postgraduate UK examinations that ChatGPT performance was tested on. The examinations encompassed the following specialties: anaesthetics (n = 2), general practice (GP; n = 1), neurology (n = 1), obstetrics and gynaecology (n = 1), ophthalmology (n = 2), orthopaedics (n = 1), surgery (n = 1), multi-specialty (n = 1). The average grade at which these examinations are sat for a UK candidate, expressed as years after graduating, is 1.92 years.

All examinations were in the format of a written exam, comprising single best answer or multiple choice questions, except one [40]. Li et al. tested ChatGPT-4 performance on Membership of the Royal College of Obstetricians and Gynaecologists (MRCOG) Part 3 virtual Objective Structured Clinical Exam (OSCE) circuit in which ChatGPT-4 outperformed two human participants with a score of 77.2%, compared with 73.7% for human participants [40].

### MRCP (UK) Part 1

The average pass marks for the previous eight MRCP Part 1 examinations were 62.0% (SD 1.76, range 58.2–64.3%). ChatGPT-3.5 took the MRCP UK Part 1 sample paper four times and scored 126, 136, 128 and 133 out of 197 giving an average percentage of 66.4% (SD 2.32, range 63.4–69.0%) which is significantly higher than the historical pass mark (P = 0.0042, 95% CI [1.74, 7.05]). This score was 4.4% above the pass mark.

ChatGPT-4 took the MRCP UK Part 1 sample paper four times and scored 164, 168, 169 and 167 out of 197, giving an average percentage of 84.8% (SD 1.10, range 83.2–85.8%) which is significantly above the historical pass mark (P = 0.0001, 95% CI [20.62, 24.97]). This score was 22.8% above the pass mark. ChatGPT-4 performance was also significantly better than ChatGPT-3.5 (P = 0.0001, 95% CI [15.25, 21.54]).

ChatGPT-4 got 16 questions incorrect across all four attempts. Of these 16 questions, 4 were categorised as factual recall and 12 as clinical reasoning. This was not found to be statistically significant compared with the proportion of factual recall and clinical reasoning questions in the MRCP Part 1 sample questions (Z = 1.0263, P = 0.30302).

### Combined analysis

ChatGPT-3.5 failed eight out of nine postgraduate UK medical exams it performed, and only achieved a mark within or above passing range in MRCP Part 1. The average score for ChatGPT-3.5 was 56% (SD 0.084, range 43%-69.7%), with an average difference between GPT-3.5 score and pass mark of -5.0%.

ChatGPT-4 passed nine out of eleven examinations it performed with an average score of 76.2% (SD 1.23, range 50%-100%), with an average difference between GPT-4 score and pass mark of +13.56%. The specialties that ChatGPT-4 failed were orthopaedics and radiology. The specific examinations that ChatGPT-4 failed were the Fellowship of the Royal College of Surgeons in Orthopaedics (FRCS (Orth)) Part 1 and the Fellowship of the Royal College of Radiologists (FRCR) Part 1 examinations, which require a minimum of 6 years and 2 years postgraduate clinical experience respectively [37,41]. Therefore, all postgraduate examinations that require 0–1 year of clinical experience were passed by ChatGPT-4.

ChatGPT-4 outperformed ChatGPT-3.5 in all examinations that were taken by both LLMs. This was reported to be significantly greater in four examinations, including MRCP Part 1 (P<0.001) [37,42,43]. The above results are outlined in Table 1.

**Table 1:** Summary of included studies with pass marks and average score of ChatGPT-3.5 and -4 included. Results from this study are included in the final row.

Author	Specialty	Exam	Grade exam sat in UK (number of years post-graduation)	Pass mark	ChatGPT-3.5%	ChatGPT-3.5 Pass/Fail	ChatGPT-4%	ChatGPT-4 Pass/Fail
Aldridge and Penders [44]	Anaesthetics	Primary FRCA	0 years		43%	Fail	63.6%	Within range of prior pass marks
Ariyaratne et al. [37]	Radiology	FRCR Part 1	2 years (must be in clinical radiology post)	75.5%			74.8%	Fail
		FRCR Part 2A		63.3%	50.8%	Fail	74.2%	Pass
Armitage [45]	GP	MRCGP	3 years				100%	Pass
Birkett et al. [38]	Anaesthetics	FRCA	0 years	71.3%	69.7%	Fail		
Fowler et al. [39]	Ophthalmology	FRCOphth Part 1	1 year	58–65%			85.7%	Pass
Ghosn et al. [42]	Radiology	FRCR Part 2A	2 years (must be in clinical radiology post)	60%	52.7%	Fail	76.5%	Pass
Giannos [46]	Neurology	UK SCE Neurology	0 years	58%	57%	Fail	64%	Pass
Li et al. [40]	Obstetrics and Gynaecology	MRCOG Part 3	4 years				77.2%	
Raimondi et al. [43]	Ophthalmology	FRCOphth Part 1	1 year	58%	55.1%	Fail		
		FRCOphth Part 2	5 years	66%	49.6%	Fail	79.1%, increased to 88.4% with prompting	Pass
Saad et al. [41]	Orthopaedics	FRCS (Orth) Part 1	6 years (4 years in orthopaedics)				50%	Fail
		FRCS (Orth) Part 2					85%	Pass
		Overall					68%	Fail
Tsoutsanis and Tsoutsanis [47]	Multi-specialty	MSRA CPS	1 year		64%	Fail		
Yiu and Lam [48]	Surgery	MRCS Part A	0 years	67–73%			85.7% (no justification), 84.3% (with justification	Pass
Vij et al.	Medicine	MRCP Part 1	1 year	58.2%-64.3%	66.4%	Pass	84.8%	Pass

## Discussion

ChatGPT-4 performed above passing level on the majority of postgraduate UK medical exams that it was tested on, with no additional training. However, these results must be interpreted with caution.

Our results demonstrate that ChatGPT-4 can answer MRCP Part 1 questions at a level that is above the historical pass mark. The MELD analysis suggest that this performance is a reflection of ChatGPT’s ability to interpret and process information rather than simply recall answers. The candidate scores for MRCP are historically variable. It has previously been shown that performance in MRCP Part 1 varies with medical school for UK applicants, and score on Professional and Linguistic Assessments Board (PLAB) examinations for international applicants [31,49]. Previous studies have demonstrated that top performers in MRCP Part 1 are applicants who attended Oxford or Cambridge University, followed by those who scored ≥35 marks above the PLAB1 pass mark (PLAB1 A1 35+) [31,49]. Oxford and Cambridge students scored an average of around +5%-10% above the MRCP Part 1 pass mark, with PLAB1 A1 35+ cohort just below this [49]. Our data indicates that ChatGPT-4 would outperform all UK medical schools by a considerable margin with an average of +22.8% above the pass mark, at least two times above the average pass mark as those who attended Oxford University. In addition, ChatGPT-3.5 would be outperformed by candidates from Oxford and Cambridge Universities but its score in the sample paper would outperform all other UK universities and international applicant scores.

Despite the evidence that ChatGPT-4 can perform well in postgraduate UK medical exams, there are still concerns with using LLMs both in medical education and its application to clinical practice. Due to their design, LLMs can produce text that sounds correct, but it cannot guarantee that it is accurate [50]. This has been demonstrated with ChatGPT, and while GPT-4 is an improvement on GPT-3.5, it is still prone to hallucination and fabrication [51]. ChatGPT has been shown to inaccurately report the content of genuine publications and when asked to generate a short literature review, 18% of GPT-4 citations were fabricated [52,53]. With many medical students and doctors using ChatGPT as an adjunct for education and clinical practice, this propensity for fabrication becomes problematic. Indeed, many papers express concerns regarding factual inaccuracies and spread of misinformation that could occur with widespread use of LLMs such as ChatGPT [54]. This propensity for misinformation is further exemplified by LLM’s poor explanation accuracy; GPT-4 demonstrated an explanation accuracy of 65.9% dropping from the correct rate of 80.5% in the FRCR Part 2A examination [42]. This suggests that while ChatGPT-4 can pass many of these postgraduate examinations, it may not be able to explain the answers correctly at a passing level. Both these factors, fabrication and reduced explanation accuracy, highlight the potential limitations for LLMs including ChatGPT as learning tools. However, the effects of fabrication and hallucination could potentially be minimised with the use of prompts which require LLMs to provide the rationale behind their clinical decision making [55,56]. Savage et al. propose a new LLM workflow in which LLMs provide a clinical reasoning rationale before their output. This offers clinicians an interpretable means to assess if the answer given by the model is true or false. Incorrect model responses are often accompanied by rationales with factual inaccuracies, rendering inaccurate answers identifiable [55]. Savage et al. suggest LLMs providing clinical reasoning rationale is achievable through diagnostic reasoning Chain-of-Though (CoT) prompting. This is a method whereby input prompts are altered to instruct a LLM to divide tasks into smaller reasoning steps. This more accurately reflects the step-by-step cognitive processes utilised by clinicians in medical practice. Despite this, only GPT-4 and not GPT-3.5 was able to imitate advanced clinical reasoning processes to arrive at an accurate diagnosis when tested with diagnostic CoT prompts, and while it was observed that GPT-4 can imitate clinical reasoning thought processes it cannot apply clinical reasoning in a comparable way to a human [55].

ChatGPT performance appeared to be enhanced with factual recall and diminished when required to apply more complex reasoning. ChatGPT performed well on shorter questions as well as those testing anatomy and pharmacology, while it performed poorly in physiology and legal or ethical questions [37,38,41]. Indeed, in MRCP Part 1, ChatGPT-4 performed worst in questions asking for further investigations, with 29.4% incorrect. This was thought to be a consequence of limited exposure, where ChatGPT is able to recall facts similar to those in the data set it was trained on, but struggles with more complex questions due to lack of higher-order thinking and limited clinical experience [41]. In contrast to this, other studies found that there was no significant difference in performance between higher and lower order questions (P = 0.816 for GPT-3.5, P = 0.427 for GPT-4) and equal proficiency when answering basic science and clinical questions [42,43]. Of the questions ChatGPT-4 got consistently incorrect on MRCP Part 1, there was no statistically significant difference between the number of incorrectly answered factual recall and complex reasoning questions (Z = 1.0263, P = 0.30302). Interestingly, ChatGPT’s performance was found to mimic performance of humans candidates, with worse results on questions humans found more challenging [39]. These differences in performance could be explained firstly due to an undertrained model, with lack of representation of data in questions where ChatGPT performed poorly [1,43]. Indeed, one study found that ChatGPT performed significantly better on general medicine than neuro-ophthalmology, suggesting lack of training on more esoteric topics [39]. However, with the improved performance of GPT-4 compared with -3.5 and a more up-to-date data set, this issue will likely become less prevalent. It could also be suggested that an insufficiency in human judgement at the initial reinforcement stages of model development, which is a significant liability of LLMs, may be additionally responsible for this change. In short this means that AI ability becomes concomitant with human ability, and would explain why ChatGPT performed worse on questions humans found more challenging [1,39,57]. This reduction in LLM’s performance in more complex reasoning could be mitigated through similar methods used to minimise hallucination. The Tree-of-Thoughts (ToT) system proposes the construction of a tree-like structure in which each node represents a partial solution in the problem-solving process. The tree comprises coherent sequences of language called “thoughts”. Each thought is then evaluated by the LLM using a deliberative reasoning process similar to that of type 2 human reasoning [56,58]. The ToT method has been applied to LLMs undertaking various complex reasoning tasks, including sudoku and crosswords, and the results suggest there cannot be non-hallucinatory, reliable human-like reasoning in LLMs without a strategy that employs a ToT, or similar system, as well as elements that mimic human-like working memory [56].

The majority of examinations discussed and analysed ChatGPT’s factual knowledge and reasoning. However, a number of medical examinations require adequate clinical skills, patient manner and professionalism–qualities required to be a safe and effective clinician [45]. It has been suggested that part of the reason ChatGPT was unable to perform well on some examination questions was due to lack of clinical experience [41]. Despite its lack of experience, ChatGPT was able to outperform human candidates at a postgraduate virtual OSCE and achieved strong marks in communication, for which empathy is essential [40]. This demonstrates ChatGPT’s ability to rapidly assemble complex clinical information into a coherent response [40]. Not only was this strong performance a surprise, but ChatGPT’s success within a field in which human interaction and communication is fundamental, has led people to suggest that we may need to change the way medical examinations are performed [43].

The strong performance of ChatGPT-4 on postgraduate UK medical exams highlights the huge potential for collaboration with AI for decision making, as well as medical education [38,44]. With the noticeable progress made from GPT-3.5 to -4 it is probable that future iterations of GPT will be even more competent [37]. Although LLMs have limitations to their utility, their ability to process complex information designed to assess high-level clinical reasoning, demonstrates the large potential AI models have as an adjunct role in medicine. It is essential for doctors to be aware of the limitations of LLMs, such as ChatGPT, so that the models can be used and applied effectively and safely into medical practice.

### Limitations

It cannot be definitively excluded that ChatGPT may have been trained on the data sets that it was tested on and was therefore simply recalling facts rather than interpreting data. However, MELD was used in this paper to show that it was unlikely that ChatGPT was trained on the MRCP (UK) Part 1 sample paper. MELD was also used in three other papers to suggest ChatGPT was not trained on the data set it was tested on [37–39].

All studies included in our SLR used proxies for the examination they were attempting to test, such as question banks or sample papers, rather than an exact examination paper. While these are likely a good representation of the examination, it is difficult to be certain of how the historical pass marks translate to sample paper and question bank performance.

Although it was possible to breakdown the question types ChatGPT got incorrect into factual recall and complex reasoning, we were unable to assess the reasoning processes which lead to the LLM making these errors. This was largely due to the style of examination questions inputted into ChatGPT, which did not necessitate a step-by-step breakdown of responses into smaller reasoning steps, unlike in CoT prompting. This means we were unable to comment on the types of reasoning errors that commonly occur leading to ChatGPT giving incorrect responses.

## Conclusions

ChatGPT-4 performed at above passing level for the majority of UK postgraduate medical examinations it was tested on, with no additional training. ChatGPT-4’s performance on MRCP(UK) Part 1 greatly outperformed the historical average performance of applicants from all medical schools.

ChatGPT-4 outperformed ChatGPT-3.5 in all examinations that they both undertook, in keeping with their respective performances on previous examinations. While improved with GPT-4, ChatGPT is prone to hallucinations, fabrications and reduced explanation accurary which could limit its potential as a learning tool. The potential for these errors are an inherent part of LLMs, thus, despite the impressive trajectory of ChatGPT from -3.5 to -4, this may always be a limitation for the medical applications of LLMs, however there is potential to minimise these effects through strategies that mimic human deliberative reasoning such as Chain-of-Thought and Tree-of-Thought search systems.

It appears that ChatGPT performed better on factual recall than higher order complex clinical scenarios, and its performance mimicked the performance of human participants, performing worse on more difficult questions. This convincing performance is likely to improve with future iterations of ChatGPT, whereby ChatGPT may become an increasingly valuable medical education resource if applied appropriately. However, it is essential that clinicians are aware of the limitations of LLMs, such as ChatGPT, if these models are to be applied safely to medical practice.

## Supporting information

S1 File Search strategies for systematic review on the capabilities of Chat-GPT in postgraduate medical assessments. (DOCX)

S2 File. PRISMA checklist (DOCX)
