# Supplementary material for: Evaluating the competency of ChatGPT in MRCP Part 1 and a systematic literature review of its capabilities in postgraduate medical assessments
Source: PLoS One. 2024 Jul 31;19(7):e0307372. doi: 10.1371/journal.pone.0307372 (PMC11290618; doi:10.1371/journal.pone.0307372)
Supplement: S1 File — (DOCX) [file pone.0307372.s001.docx]

**Search Strategies**

**Medline**

**Ovid MEDLINE(R) ALL <1946 to January 22, 2024>**

**Date: 23/01/2024**

1. exp Artificial Intelligence/ 187876
2. (artificial intelligence$ or artificial general intelligence$ or artificial narrow intelligence$ or computational intelligence$ or artificial superintelligence$ or AI-complete or AI-powered or explainable AI or interpretable AI or deep learning).ti,ab,kw,kf. 94663
3. Al.ti. 6721
4. (language model$ or LLM$1 or natural language processing or NLP$1).ti,ab,kw,kf. 13767
5. ((AI or "computer assisted " or "computer aided" or deep learning) adj2 algorithm$).ti,ab,kw,kf. 7249
6. (conversational agent$ or conversational AI or chat bot$ or chatbot$ or "Generative Pre trained Transformer 4" or "GPT 4" or ChatGPT or chat gpt).ti,ab,kw,kf. 3737
7. or/1-6 247114
8. medical student/ or medical education/ or medical school/ or educational measurement/
9. (medical adj2 (student$ or educat$ or postgraduate$ or post grad$ or school$)).ti,ab,kw,kf. 144452
10. or/8-9 224877
11. (exam$ or assessment$).ti,ab,kw,kf. 5070561
12. 10 and 11 57759
13. (Clinical Problem Solving adj2 paper$).ti,ab,kw,kf. 5
14. (Multi Specialty Recruitment Assessment or (MSRA adj2 (exam$ or assessment$))).ti,ab,kw,kf.
15. (royal college$ adj1 (membership$ or fellowship$)).ti,ab,kw,kf. 10
16. (MCEM or MRCEM or MRCOG or MFOM or MRCOphth or MRCPCH or MRCP or "MRCP(UK)" or MRCPysch or MFPH or MRCS or FRCA MCG or FCEM or FRCEM or FRCOphth or FRCPath or FRCPsych or FRCR or FRCS).ti,ab,kw,kf. 4807
17. or/13-16 4845
18. 12 or 17 62438
19. 7 and 18 668
20. limit 19 to english language 660

**Embase**

**Embase <1974 to 2024 January 2022>**

**Date: 23/01/2024**

1. exp artificial intelligence/ 95147
2. artificial intelligence$ or artificial general intelligence$ or artificial narrow intelligence$ or computational intelligence$ or artificial superintelligence$ or AI-complete or AI-powered or explainable AI or interpretable AI or deep learning).ti,ab,kw,kf. 110342
3. AI.ti. 8663
4. (language model$ or LLM$1 or natural language processing or NLP$1).ti,ab,kw,kf. 15940
5. ((AI or "computer assisted " or "computer aided" or deep learning) adj2 algorithm$).ti,ab,kw,kf. 9752
6. (conversational agent$ or conversational AI or chat bot$ or chatbot$ or "Generative Pre trained Transformer 4" or "GPT 4" or ChatGPT or chat gpt).ti,ab,kw,kf. 3542
7. or/1-6 177350
8. medical student/ or exp medical education/ 417244
9. (medical adj2 (student$ or educat$ or postgraduate$ or post grad$ or school$)).ti,ab,kw,kf. 173251
10. or/8-9 459551
11. (exam$ or assessment$).ti,ab,kw,kf. 6636524
12. 10 and 11 96948
13. (Clinical Problem Solving adj2 paper$).ti,ab,kw,kf. 4
14. (Multi Specialty Recruitment Assessment or (MSRA adj2 (exam$ or assessment$))).ti,ab,kw,kf. 29
15. (royal college$ adj1 (membership$ or fellowship$)).ti,ab,kw,kf. 9
16. (MCEM or MRCEM or MRCOG or MFOM or MRCOphth or MRCPCH or MRCP or "MRCP(UK)" or MRCPysch or MFPH or MRCS or FRCA MCG or FCEM or FRCEM or FRCOphth or FRCPath or FRCPsych or FRCR or FRCS).ti,ab,kw,kf. 9862
17. or/13-16 9903
18. 12or17
19. 7 and 18 847
20. Limit 19 to english language 836

**Cochrane**

**Cochrane Central Register of Controlled Trials (CENTRAL) and Cochrane Database of Systematic Reviews (CDSR)**

**Date: 23/01/2024**

1. MeSH descriptor: [Artificial Intelligence] explode all trees 2986
2. (("artificial intelligence" or "artificial general intelligence" or "artificial narrow intelligence" or "computational intelligence" or "artificial superintelligence" or "AI-complete" or "AI- powered" or "explainable AI" or "interpretable AI" or "deep learning"):ti,ab,kw 3041
3. (AI):ti 766
4. (language NEXT model* or LLM or "natural language 86 processing" or NLP):ti 86
5. ((AI or "computer assisted" or "computer aided" or deep 156 learning) NEAR/2 algorithm*):ti 156
6. (conversational NEXT agent* or "conversational AI" or chat NEXT bot* or chatbot* or "Generative Pre trained Transformer 4" or "GPT 4" or ChatGPT or "chat gpt"):ti 171
7. {OR #1-#6} 5970
8. MeSH descriptor: [Students, Medical] this term only 1561
9. MeSH descriptor: [Education, Medical] explode all trees 4119
10. MeSH descriptor: [Schools, Medical] this term only 97
11. MeSH descriptor: [Educational Measurement] this term only 2087
12. (medical NEAR/2 (student$ or educat* or postgraduate* or 559 post NEXT grad*)):ti
13. {OR #8-#12} 6272
14. exam* or assessment* 556242
15. #13 and #14 3093
16. "clinical problem solving" NEAR/2 paper* 0
17. ("Multi Specialty Recruitment Assessment" or (MSRA NEAR/2 0 (exam* or assessment*))):ti,ab,kw 0
18. (royal NEXT college* NEAR/1 (membership* or 0 fellowship*)):ti,ab,kw 0
19. (MCEM or MRCEM or MRCOG or MFOM or MRCOphth or MRCPCH or MRCP or "MRCP(UK)" or MRCPysch or MFPH or MRCS or FRCA MCG or FCEM or FRCEM or FRCOphth or FRCPath or FRCPsych or FRCR or FRCS):ti,ab,kw 318
20. {OR #16-#19} 318
21. #15 or #20 3411
22. #7 AND #21 27
